# Maleimide–Thiol Linkages Alter the Biodistribution of SN38 Therapeutic Microbubbles Compared to Biotin–Avidin While Preserving Parity in Tumoral Drug Delivery

**DOI:** 10.3390/pharmaceutics16030434

**Published:** 2024-03-21

**Authors:** Nicola Ingram, Radwa H. Abou-Saleh, Amanda D. Race, Paul M. Loadman, Richard J. Bushby, Stephen D. Evans, P. Louise Coletta

**Affiliations:** 1 Leeds Institute of Medical Research, Faculty of Medicine and Health, St James’s University Hospital, Beckett Street, Leeds LS9 7TF, UK; 2 School of Physics and Astronomy, Sir William Henry Bragg Building, University of Leeds, Leeds LS2 9JT, UK; r.h.saleh@gu.edu.eg (R.H.A.-S.);; 3 Physics Department, Faculty of Science, Galala University, Galala 43711, Egypt; 4 Department of Physics, Mansoura University, Mansoura 35516, Egypt; 5 Institute of Cancer Therapeutics, University of Bradford, Bradford BD7 1DP, UKp.m.loadman@bradford.ac.uk (P.M.L.)

**Keywords:** microbubbles, maleimide, biotin, therapy, preclinical, drug delivery

## Abstract

Therapeutic microbubbles (thMBs) contain drug-filled liposomes linked to microbubbles and targeted to vascular proteins. Upon the application of a destructive ultrasound trigger, drug uptake to tumour is improved. However, the structure of thMBs currently uses powerful non-covalent bonding of biotin with avidin-based proteins to link both the liposome to the microbubble (MB) and to bind the targeting antibody to the liposome–MB complex. This linkage is not currently FDA-approved, and therefore, an alternative, maleimide–thiol linkage, that is currently used in antibody–drug conjugates was examined. In a systematic manner, vascular endothelial growth factor receptor 2 (VEGFR2)-targeted MBs and thMBs using both types of linkages were examined for their ability to specifically bind to VEGFR2 in vitro and for their ultrasound imaging properties in vivo. Both showed equivalence in the production of the thMB structure, in vitro specificity of binding and safety profiles. In vivo imaging showed subtle differences for thMBs where biotin thMBs had a faster wash-in rate than thiol thMBs, but thiol thMBs were longer-lived. The drug delivery to tumours was also equivalent, but interestingly, thiol thMBs altered the biodistribution of delivery away from the lungs and towards the liver compared to biotin thMBs, which is an improvement in biosafety.

## 1. Introduction

Contrast-enhanced ultrasound is a powerful tool for diagnosis of disease and has been incorporated into clinical practice in such diverse areas as diagnosis of hepatocellular carcinoma, kidney cysts and endothelial leakage in the aorta. Contrast agents typically consist of a lipid/protein membrane surrounding a perfluoro gas. They are approximately 2 μm in diameter and are restricted to the vascular system where they strongly reflect acoustic waves [1]. The use of microbubbles (MBs) as a therapeutic vehicle is now being explored. To this end, therapeutic antibodies, drug-loaded liposomes and genetic material have all been conjugated to or co-injected with MBs and several clinical trials have been initiated (see [2] for an up-to-date review on the topic).

Our laboratory is currently studying the use of MBs for cancer-targeted drug delivery using liposomally encapsulated drugs attached to the MBs which are also targeted to tumour vasculature via the use of antibodies. The use of the avidin–biotin binding system to attach such structures is widely used due to its extreme stability in different pH environments, temperatures and solvents and its very strong non-covalent bonding (K_d_ = 10^−15^ M at pH 5.0) [3]. In addition, one avidin can bind up to four biotin molecules, thus allowing multiple attachments. However, many groups have also explored the use of compounds that can form bonds with sulfhydryls (–SH, also called thiols). These are most commonly used with proteins, as sulfhydryls occur in proteins in the side-chain of cysteine amino acids in disulphide bonds that do not tend to disrupt protein structure or function when reduced. These thiolated proteins can then be reacted with maleimide, via a reversible thioether linkage with an equilibrium constant of ~10^9^ M^−1^ [4], pyridyldithiopropionate (PDP) or phosphothioethanol (PtD), via disulphide exchange reactions, among others. These alternative linkages are used in FDA-approved antibody–drug conjugates such as Adcentris^®^, Kadcyla^®^ and Sacituzumab Govitecan, a Trop2-targeting antibody linked to the active molecule of irinotecan, SN38, which is being fast-tracked for FDA approval.

Biotin–avidin linkages have been used in multiple therapeutic microbubble (thMB) delivery platforms, including the delivery of anti-angiogenic inhibitors [5], siRNAs on nanobubbles (nanometre-sized bubbles) [6], drug-loaded liposomes with additional molecular targeting [7,8] or combined therapies [9]. Maleimide–thiol linkages have similarly been used for liposomal drug delivery [10,11] or to detect and kill circulating tumour cells [12] and for DNA delivery [13]. In addition, these linkages were used to generate diagnostic MBs for targeted imaging of E-Selectin [14,15], secreted frizzled-related protein 2 [16] or CD146, a co-receptor for VEGFR2 [17]. 

However, although these studies have shown promising results pre-clinically, to our knowledge, no study has directly compared whether there is an advantage or disadvantage of each linkage in terms of targeting, ultrasound imaging properties or drug delivery or whether they show parity. Therefore, the aim of this study was to define which liposome–MB linkage system was most suited to each of these applications.

## 2. Methods

### 2.1. Preparation of Thiolated Antibodies

Anti-mouse VEGFR2 antibody (clone Avas12a1, eBiosciences, San Diego, CA, USA) or its isotype control (anti-mouse IgG2a) were concentrated using a centrifugal filter (Amicon^®^ Ultra Centrifugal Filter, 3 kDa MWCO, Merck, Rahway, NJ, USA) following the manufacturer’s instructions. The optical density at 280 nm was measured using a NanoDrop One spectrophotometer to determine the concentration of the antibodies after concentration. Next, 100 molar excess Traut’s reagent (ThermoFisher Scientific, Waltham, MA, USA) freshly dissolved in 5 mM EDTA, pH 8 in phosphate-buffered saline (PBS) was incubated with the antibodies in the dark at room temperature for one hour with agitation. Excess Traut’s reagent was then removed by passing the antibodies through a protein desalting spin column (Amintra, Expedeon, Cambridge, UK). Verification of thiolation was carried out using Ellman’s reagent (ThermoFisher Scientific) against a cysteine (ThermoFisher Scientific) standard curve diluted in 1 mM EDTA, pH 8.0 in PBS.

### 2.2. Preparation of Microbubbles

DPPC:DSPE-PEG _2000_-biotin (95:5 molar ratio) or DPPC:DSPE-PEG _2000_:DSPE-PEG _2000_-maleimide (95:3:2 molar ratio) (all Avanti Polar Lipids, Merck) were dried under nitrogen. Lipids were then dissolved in buffer (1% *v*/*v* glycerol, 0.4% *w*/*v* NaCl in dH_2_O) to 2 mg/mL in a sonicating waterbath and MBs were prepared using a custom microfluidics set-up as described previously [18] incorporating a C_4_F_10_ gas core and C_6_F_14_ liquid [19]. Quantitation of MB concentration and number was carried out by taking pictures of the MBs under ×60 magnification on a Nikon Eclipse Ti microscope (Nikon Europe B.V., Amstelveen, The Netherlands) and quantitation using a custom macro on ImageJ, version 1.53, as described previously [18].

### 2.3. Preparation of Drug-Loaded Liposomes

SN38 was encapsulated in liposomes as described previously. Briefly, for biotin-containing liposomes, DSPC:Cholesterol:DSPE-PEG _2000_-biotin (57:39:4 molar ratio) (all Avanti Polar Lipids) were combined with cardiolipin (Merck) at 11% of the total lipid mass [20]. For thiol-containing liposomes, DSPC:Cholesterol:DPPTE:DSPE-PEG _2000_ (57:39:3:2 molar ratio) were combined with cardiolipin at 11% of the total lipid mass. Lipids were dried under nitrogen for 1 h then under vacuum overnight. The lipid mixtures were dissolved in 0.4 mg/mL SN38 (Merck) in alkaline buffer pH 11.2 to a lipid concentration of 42 mg/mL. Liposomes were extruded through a 200 nm filter with a mini-extruder at 65 °C. To the thiol-containing liposomes, 20 μL of 2 mg/mL TCEP was added per 500 μL of liposomes. After 15 min incubation, the liposomes were freeze-dried in aliquots. Liposomes were rehydrated in acetate buffer pH 2 and filtered through a 200 nm syringe filter to remove any unencapsulated SN38. 

### 2.4. Characterisation of Liposomes

Quantitation of SN38 encapsulated within liposomes was carried out by measuring the optical density at 383 nm of liposomes in methanol (UV-Vis, Agilent Cary 3500, Santa Clara, CA, USA) and comparing to known quantities of free SN38 in methanol to generate a standard curve for interpolation. The mean diameter and concentration of the liposomes were measured using by nanoparticle tracking analysis on a NanoSight NS300 (Malvern Analytical, Westborough, MA, USA) with the flow cell. Screen gain was set to 1 and the camera level to 14. Five 60 s loops were acquired at a flow rate of 30 μL/min, and the detection threshold was set to 5.

### 2.5. Preparation of Therapeutic Microbubbles, thMBs

Thiolated antibodies and liposomes were prepared as above. For the biotinylated versions, 100 μL of liposomes was incubated with 10 μL of 2.5 mg/mL neutravidin for 20 min at room temperature and then incubated with 1 mL of MB lipids for the same time before making MBs as described. Next, 0.1 μg of biotinylated antibodies (biotin-conjugated version of Avas12a1 or isotype, eBiosciences) was then added per 10^7^ microbubbles prepared and incubated at room temperature for 20 min to allow binding to the available avidin sites before use [7]. For the thiolated versions, 100 μL of liposomes was incubated with 1 mL of MB lipids for at least 30 min before making MBs, and then 0.1 μg of freshly thiolated antibody per 10^7^ MBs was incubated for a further 30 min before use. Binding of liposomes and antibodies could equally be carried out post-MB manufacture, where typically 2 × 10^8^ MBs were bound to 100 μL of liposomes (approximately 6 × 10^11^ liposomes).

### 2.6. Immunofluorescence

SVR cells (mouse endothelial cells expressing VEGFR2, ATCC^®^ CRL-2280^™^) were grown on glass coverslips and fixed in 100% methanol. After blocking with Antibody diluent (Invitrogen, Carlsbad, CA, USA), thiolated or un-thiolated anti-VEGFR2 was incubated at 0.2 mg/mL for 1 h at room temperature. Following extensive washing, the secondary antibody (FITC-conjugated goat anti-rat, Merck) was incubated at 1:100 dilution for 30 min in the dark. After further washing, the coverslips were mounted using Prolong Gold + DAPI (Invitrogen).

For imaging the liposome-loaded MBs, Texas Red^®^ 1,2-dihexadecanoyl-sn-glycero-3-phosphoethanolamine, triethylammonium salt (Texas Red^®^ DHPE, Avanti Polar Lipids, Alabaster, AL, USA) was incorporated at 0.1 mole % to the liposomes. The generation of thMBs was carried out as described above except that the liposomes were incubated with the MB lipids overnight before making MBs. MBs with attached liposomes were placed on a slide with a coverslip raised on 50 μm-thick acetate and imaged using a Nikon Eclipse microscope using a ×60 objective.

### 2.7. Flow Cytometry

For quantification of MBs loaded with liposomes, Atto 647N DOPE was incorporated at 0.05 mole % into the liposome and 0.05 mole% Atto 488 DOPE was incorporated into the shell of the MB. The generation of thMBs was carried out as described above and diluted to 1 × 10^7^ MBs per mL before running in triplicate on a CytoFLEX S flow cytometer (Beckman Coulter, Brea, CA, USA) and analysed using CytExpert software, version 2.4, (Beckman Coulter). The settings for FSC and SSC were gated using PBS only and Megamix-Plus FSC beads (catalogue number:7802 Biocytex, Marseille, France) were to exclude anything less than 500 nm. A total of 50,000 events were collected within this gate and the populations were analysed for green and red fluorescence positivity compared to MBs with no liposomes attached.

### 2.8. Quantitation of Microbubble Targeting Efficiency In Vitro

SVR cells were plated in μ-Slide VI^0.4^ (ibidi, Thistle Scientific, Uddingston, UK), and 10^7^ MBs were flowed across using a syringe driver as described previously [18]. After extensive washing, brightfield images were taken and the number of MBs attached to cells was counted along with the total number of cells in the field of view. The frequency of MB attachment was also determined by counting the total number of cells in the field of view with 1, 2, 3, 4, or 5 or more MBs attached.

### 2.9. In Vivo Lifetime Assay

CD1 nude male mice aged 7–9 weeks (Charles River Laboratories, Wilmington, MA, USA) were housed in individually ventilated cages with free access to food and water on a 12 h day/night cycle. All experiments were performed following local ethical approval and in accordance with the Home Office Animal Scientific Procedures Act (1986). Mice were euthanised by cervical dislocation at the end of the experiments.

Mice were anaesthetised using isofluorane and a 27-gauge mouse tail vein needle with infusion tubing (SAI Infusion Technologies, Lake Villa, IL, USA) was inserted. The aorta and inferior vena cava were imaged caudally on the abdomen with the mouse in the supine position to reduce artefacts from respiration. Using the RMV 704 transducer (40 MHz) attached to a Vevo 770 high-frequency ultrasound machine (FUJIFILM VisualSonics, Toronto, ON, Canada), Pulse Wave Doppler imaging was used to confirm the pulsatile flow in the aorta. A syringe driver was used to inject 3 × 10^7^ thMBs in 100 μL volume at 0.6 mL/min. Cine loops of the injection and for the next 15 min with one-minute gaps between each cine loop were recorded in contrast mode at 50% power.

### 2.10. Analysis of Microbubble Lifetimes

Using the VisualSonics Vevo 770 software, version 2.0.0, the Time Intensity Curve (TIC) of each injection was obtained by choosing the first one hundred frames of the cine loop (before the MBs were injected) as the reference frames. The loop was then processed and a circular region of interest of 0.12 mm^2^ was drawn within the aorta avoiding the walls of the vessel. The resulting intensity within that region was used to plot a TIC and fitted to a Boltzmann curve to determine the following parameters: peak enhancement (maximum intensity–minimum intensity), time to peak (time to reach maximum intensity from minimum intensity) and wash-in rate (slope of the ascending curve). The subsequent cine loops were processed using the same pre-microbubble reference frames and region of interest. These were plotted alongside the TIC and fitted with a LogNormal curve from 5 s to 1000 s. Linear regression on the fitted curve from 200 to 600 s was used to generate the wash-out rate (slope of the descending curve). The area under the curve (AUC) was calculated from the LogNormal fit using the minimum intensity as baseline. The lifetime of the MB population (FWHM, Full Width at Half Maximum intensity) was extrapolated from the curve as the time from which the intensity reaches half of the maximum intensity upon injection of MBs (taking into account the minimum intensity baseline) until the intensity of those MBs has decayed to half the maximum intensity again.

### 2.11. Analysis of In Vivo Physiology and Comparison to Lifetime Parameters

For each mouse, the MB injection loop and final imaging loop (9th) were used to generate the mean heart rate and respiration rate at the beginning and end of the imaging session. The distance from the middle of the aorta to the skin line was also measured on the injection loops. Using the pulse-wave Doppler loops, the peak positive and peak negative velocity were determined, as well as the Velocity Time Interval (VTI—how far the blood travels within a flow period), using the measurement tools provided in the software.

### 2.12. Cytotoxicity Assay

Mycoplasma-free SW480 and SW620 cells were originally obtained from ATCC and authenticated in-house by single tandem repeat profiling. Cells were grown in DMEM (Merck, high glucose) supplemented with 10% (*v*/*v*) FCS (ThermoFisher), 1% (*v*/*v*) L-glutamine (ThermoFisher). For testing the cytotoxicity of each SN38-loaded thMB type, 10,000 cells were plated per well in a 96-well plate. The following day, serial dilutions of the thMBs were added to the cells. After a further 72 h, 10 μL CCK8 (Abcam, Bristol, UK) was added per well and incubated for 3 h. The absorbance at 460 nm was read using a plate reader (SpectraMax M2, Molecular Devices, San Jose, CA, USA). The mean absorbance from 2 wells containing cell culture medium with thMB alone (no cells) was subtracted from the mean of 4 wells containing cells and thMBs. Three biological replicates were carried out. Results are expressed as a % of vehicle and fitted with a log (inhibitor) vs. response variable slope (four parameters, SW480 cells; or three parameters, SW620 cells) using least squares regression to obtain the IC_50_.

### 2.13. Haemolysis Assay

Blood was collected after cervical dislocation by cardiac puncture from five 10-week-old male C57BL/6 mice into a K3 EDTA containing paediatric blood tube (Greiner, Slušovice, Czech Republic). The blood was centrifuged at 500× *g* for 5 min at 4 °C. The plasma was removed and the cells resuspended gently to the original total volume with 150 mM NaCl. This washing step was repeated twice more before the red blood cells were diluted 1:50 in PBS. Red blood cells incubated with a final concentration of 1% (*v*/*v*) Triton-X100 was used as a positive control (100% lysed) and red blood cells incubated with PBS was used as the negative control (0% lysed). An amount of 190 μL of red blood cells was incubated with thMBs (neat or diluted with PBS) on an orbital shaker at 100 rpm for 1 h at 37 °C in triplicate for each. After centrifuging for 5 min at 500× *g* to pellet intact red blood cells, the supernatant containing any lysed red blood cells taken for absorbance measurement at 540 nm.

### 2.14. Drug Delivery

Ten CD1 nude female mice aged 8 weeks (Charles River Laboratories) were injected subcutaneously with 1 × 10^7^ SW620 cells to the right hind flank. Tumours were allowed to grow for 14 days before typically a single 100–150 μL amount of VEGFR2-targeted thMBs (5 × 10^7^ thMBs) was injected via the tail vein followed 4 min later by a MB destruction ultrasound pulse [7].

Mice were euthanized by cervical dislocation and the tumour and tissues were collected 1 h after the ultrasound pulse. The amount of SN38 and its glucuronide was quantitated by liquid chromatography tandem mass spectrometry as described previously [7].

### 2.15. Statistical Analysis

Statistical analyses were carried out using GraphPad Prism software version 10.1.1. The sample number and statistical test used along with the significance value are denoted in each figure legend.

## 3. Results

**Figure 1:**
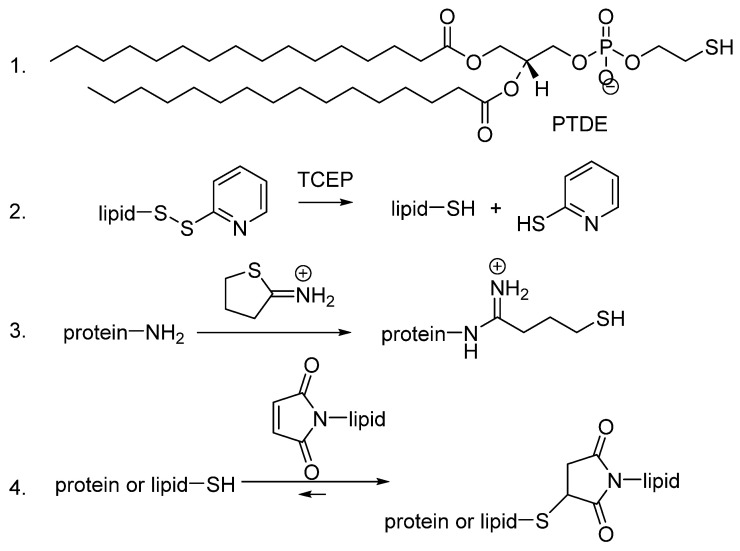
Thiol and maleimide Functionalisation. Thiol functionality of lipids was provided either (1) by incorporating PDTE into the phospholipid layer or (2) by incorporating a PDP lipid which was reduced with TCEP. (3) Thiol functionality of proteins was achieved by reacting with Traut’s reagent. (4) Linkage of the these was achieved by a reversible reaction of the thiol functionalised phospholipids or proteins with lipids that were functionalised with maleimide, which strongly favours the generation of the thiol-maleimide linkage.

Therapeutic MBs consist of drug-loaded liposomes linked to MBs with both of these structures (due to biotin–avidin linkages) attached to antibodies [7]. In order to replace the biotin–avidin link between liposome, MB and antibody, the use of maleimide-containing lipids was explored. The chemistry to allow these linkages is described in Figure 1. Commercially available PDTE or TCEP-reduced PDP phospholipids can be reacted with maleimide-containing lipids (step 2). Antibodies (proteins) can be thiolated as shown in step 3, and these could then be reacts favourably with the maleimide-containing lipids to combine the complete structure (step 4). 

**Figure 2:**
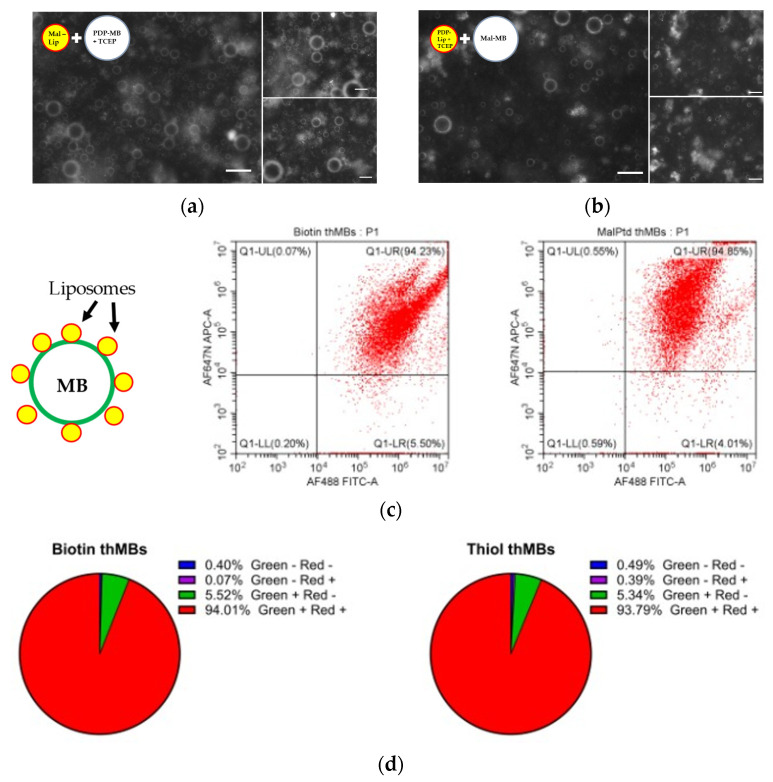
Liposomes can be bound to MBs using maleimide–thiol linkages to the same degree as biotin–avidin linkages. (**a**,**b**) Linkages using either maleimide liposomes with PDP-containing MBs or PDP liposomes with maleimide-containing MBs are feasible. The fluorescence detected around the MBs shows loading of the liposomes on the MB surface. Scale bar denotes 10 μm. (**c**) Biotin and maleimide MBs were generated with green fluorescent lipid in their shell and the SN38-encapsulating liposome (biotin or PtD) was generated with red fluorescent lipid in their shell. Example flow cytometry plots gated on the MB population show the fluorescence from biotin liposome-loaded MBs (left) or thiol liposome-loaded MBs (right). The pie charts show the percentage of all MBs with liposomes bound to them (green +, red +) as determined by flow cytometry (**d**).

To examine the use of step 2 to load liposomes onto MBs, fluorescently labelled, liposome-loaded MBs were imaged using confocal microscopy and quantitated by flow cytometry (Figure 2). Very low amounts of the reducing agent TCEP (14 mM) were used to bind PDP-containing lipids to maleimide groups. It did not seem to matter in terms of loading onto MBs whether the maleimide was part of the MB structure or liposome structure as both showed equal fluorescence around the MB (Figure 2a,b). With further investigation, it was found that PtD-lipids were slightly more stable upon reconstitution and therefore were subsequently used in place of PDP. MBs with biotin–avidin linkages were compared to maleimide-containing MBs bound to PtD-containing liposomes. The microbubbles were labelled with a green fluorescent lipid and the liposomes with a red fluorescent lipid, as shown in Figure 2c. Flow cytometry was used to quantitate the percentage of MBs (green) that were bound to liposomes (red). Both types of microbubble showed that around 95% of microbubbles were conjugated to liposomes, with around 5% of microbubbles not binding any liposomes.

**Figure 3:**
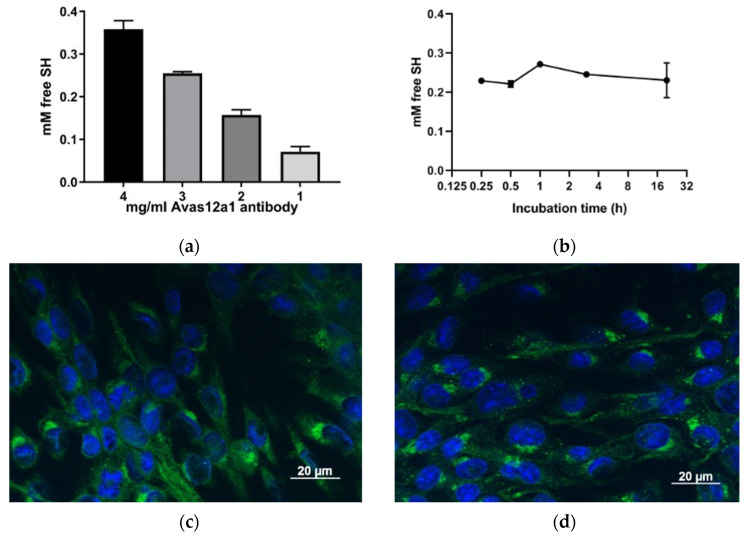
Antibodies can be thiolated using Traut’s reagent and retain their antigen recognition properties. The anti-mouse VEGFR2 antibody, Avas12a1 was thiolated using Traut’s reagent. Ellman’s assay quantified the amount of free sulfhydryl with decreasing concentrations of input antibody (**a**) and over incubation time with the Traut’s reagent using 3 mg/mL antibody (*n* = 3, mean and standard deviation are shown) (**b**). Mouse endothelial SVR cells were incubated with thiolated (**c**) or non-thiolated (**d**) mouse anti-VEGFR2 antibody to examine any difference in staining patterns that would indicate disruption of VEGFR2 recognition.

For step 3, simple thiolation of antibodies using Traut’s reagent was examined to see if the antibodies retained their antigen recognition and could be used to target MBs. Using Ellman’s Assay, the amount of thiol groups was determined and showed a linear relationship with input antibody concentration (Figure 3a). This simple thiolation was shown to be complete within 1 h incubation with Traut’s reagent, and this was subsequently used for all experiments (Figure 3b). Immunofluorescence using thiolated (Figure 3c) and non-thiolated (Figure 3d) antibody on VEGFR2-expressing SVR cells were visualised, and these showed no difference in intensity or staining pattern.

**Figure 4:**
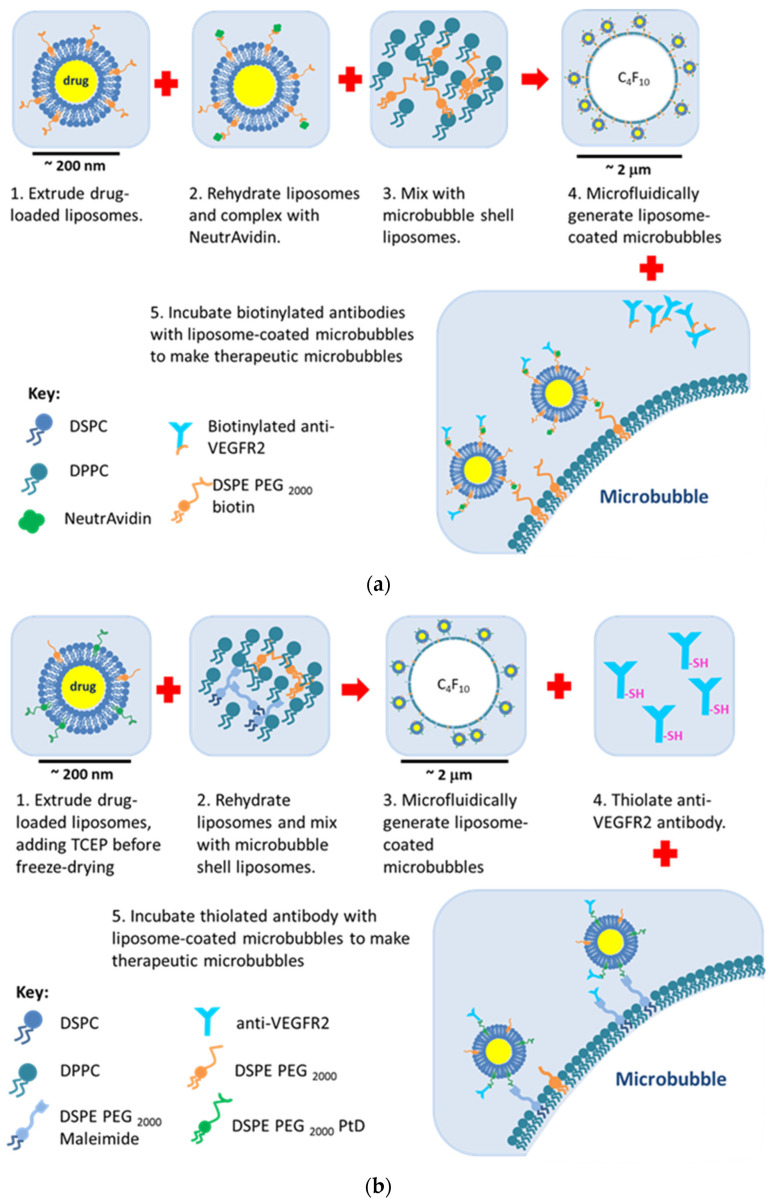
Schematic of the generation of thMBs using biotin–avidin and maleimide–thiol linkages. (**a**) Drug-loaded liposomes incorporating biotinylated lipids are generated by thin film hydration and extrusion. Liposomes are rehydrated after freeze drying and incubated with NeutrAvidin. Liposomes are then mixed with MB shell lipids in buffer. Microfluidics are used to generate liposome-loaded gas-filled MBs. These are incubated with commercially available biotinylated antibodies to generate thMBs. (**b**) Drug-loaded liposomes incorporating PtD lipids are generated by thin film hydration and extrusion and a small amount of TCEP is added before freezing. Liposomes are rehydrated after freeze drying and mixed with MB shell lipids in buffer. Microfluidics are used to generate liposome-loaded gas-filled MBs. Separately, the antibody is thiolated using Traut’s reagent. These antibodies are incubated with the liposome-loaded MBs to generate thMBs.

The steps involved to use both biotin and maleimide–thiol binding regimes to produce thMBs are shown in Figure 4. The liposome shell and the MB shell both contain biotinylated phospholipids which are bound to each other via an avidin bridge (Figure 4a). Commercially biotinylated antibodies are able to bind to the liposomes using free avidin sites. In the second case, typically commercially available PDTE was used to generate drug-loaded liposomes and a very small amount of TCEP was added to prevent hydrolysis. Commercially available maleimide-containing lipids were used in the MB shell and the thiol-functionalised liposomes were complexed with the maleimide-containing MBs. Antibodies functionalised via Traut’s reagent were then complexed to the liposome-loaded MBs (Figure 4b). 

**Figure 5:**
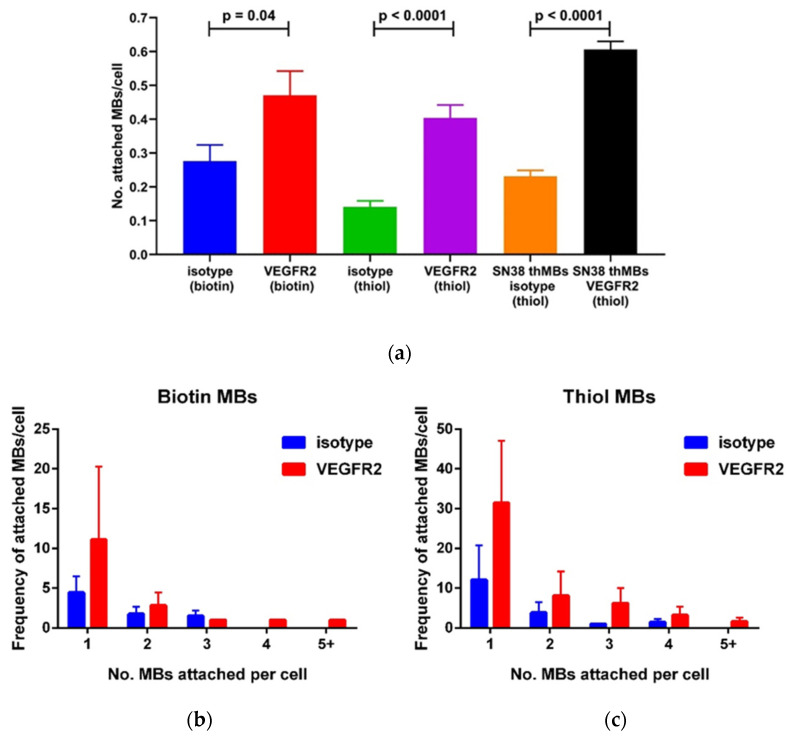
Thiolated antibodies on MBs or thMBs show good targeting specificity in vitro. (**a**) VEGFR2- or isotype-targeted MBs were bound to mouse SVR endothelial cells under fluid flow. Biotin–avidin linkages and maleimide–thiol linkages for both VEGFR2-targeted MBs and thMBs were compared to isotype, unpaired *t*-test, two-tailed). *n* = 3 experimental replicates; mean and standard deviation are shown. The frequency of single or multiple targeted MBs bound per cell was plotted for isotype or targeted antibodies with biotin linkages (**b**) or thiol linkages (**c**).

The targeting specificity of both antibody-targeted MBs alone and thMBs was determined under flow and compared to the biotinylated version to determine whether this property had been affected by thiolation. MBs bearing either biotinylated or thiolated VEGFR2 or isotype control antibodies were flowed over SVR cells. After washing off the unbound MBs, the number of MBs attached to cells was counted from microscopy images of the cells and MBs (Figure 5a). The targeting ratio (number of VEGFR2-targeted MBs attached to cells/number of isotype-targeted MBs attached to cells) was 1.7 for biotinylated MBs, 2.9 for thiolated MBs and 2.6 for thiolated thMBs. This indicates the targeting specificity in vitro was not compromised by thiolation and even slightly better than biotinylated MBs. The pattern of MB binding to cells between biotinylated and thiolated antibodies was also similar (Figure 5b,c). The majority of cells counted had a single MB attached. The frequency decreased with increasing MBs attached with fewer MBs bound if isotype control antibodies were used to target the MBs compared to VEGFR2-antibodies. The frequency with the thiolated antibodies (Figure 5c) was higher than biotin (Figure 5b) as there were more cells in each field of view.

Both biotinylated and thiolated MBs have been used for multiple drug delivery applications; however, they have never been compared directly for drug delivery efficacy. Therefore, drug loading efficiencies and thMB generation were examined. Table 1 shows the characteristics and drug loading capacity of each type of liposome and Table 2 shows the liposome-loaded MBs generated. No significant differences were observed, although it was noted that liposomes containing PtD phospholipids were easier than biotin-functionalised phospholipids to both extrude and filter through the 0.2 μm syringe filter after rehydration. This may account for the slightly lower amount of encapsulated SN38 found with biotinylated liposomes if unencapsulated particulate SN38 was being filtered out.

**Figure 6:**
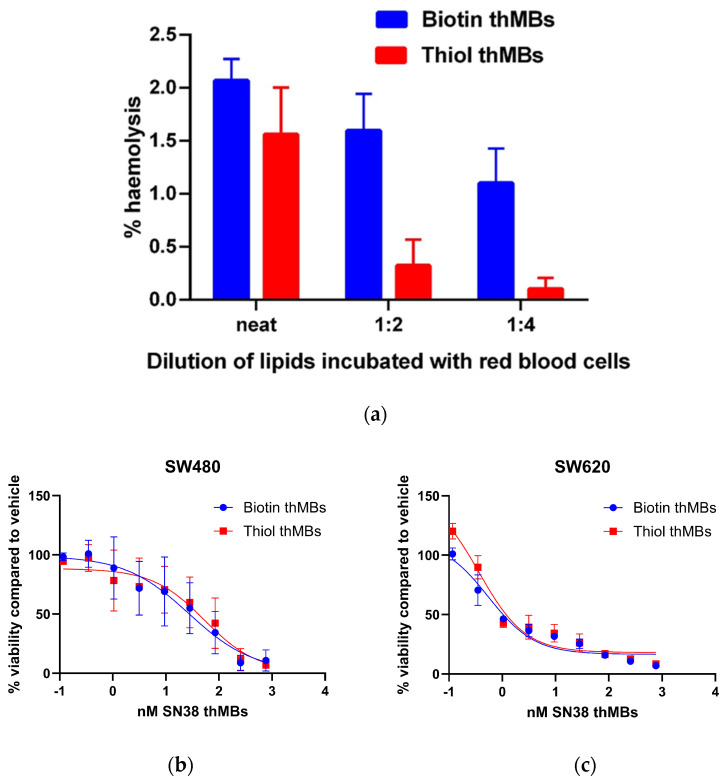
Significant haemolysis was not observed with either biotinylated or thiolated MB architectures and showed no significant differences in cytotoxicity in vitro. (**a**) Liposome-loaded MBs were incubated with red blood cells from 5 mice in triplicate. The amount of haemolysis observed was quantitated compared to a positive control (addition of Triton X-100-treated red blood cells). *n* = 3, mean and standard deviation are shown. Cells were incubated with SN38 liposome-loaded MBs and the reduction in cell viability was measured in SW480 (**b**) and SW620 (**c**) cells. The graph shows the mean and standard deviation in cell viability compared to vehicle, *n* = 3.

Before injection of these MB structures in vivo, it was necessary to determine whether there was any toxicity that may be associated with injection due to the change in manufacture. Red blood cells isolated from mice were incubated with either biotinylated or thiolated thMB structures. Neither structure generated any significant lysis of the red blood cells (Figure 6a) since anything less than 2% is considered non-haemolytic [21,22]. Both structures were therefore considered safe for injection. To test the equivalence in terms of delivery of chemotherapeutic effect, SN38 liposome-loaded MBs were incubated with cells and the cytotoxicity measured after 72 h (Figure 6b,c). Using two colorectal cancer cell lines, both the biotin and the thiol SN38 liposome-loaded MBs showed no significant differences in their reduction in cell viability. An IC_50_ of 27.4 nM (4.1 to 67.9 nM, 95% confidence interval, CI) for biotin and 53.5 nM (13.6 to 114.2 nM, 95% CI) for thiol were calculated for SW480 cells. For SW620 cells, calculated IC50 values were 0.55 nM (0.25 to 1.2 nM, 95% CI) and 0.37 nM (0.19 to 0.67 nM 95% CI) for biotin and thiol thMBs, respectively.

**Figure 7:**
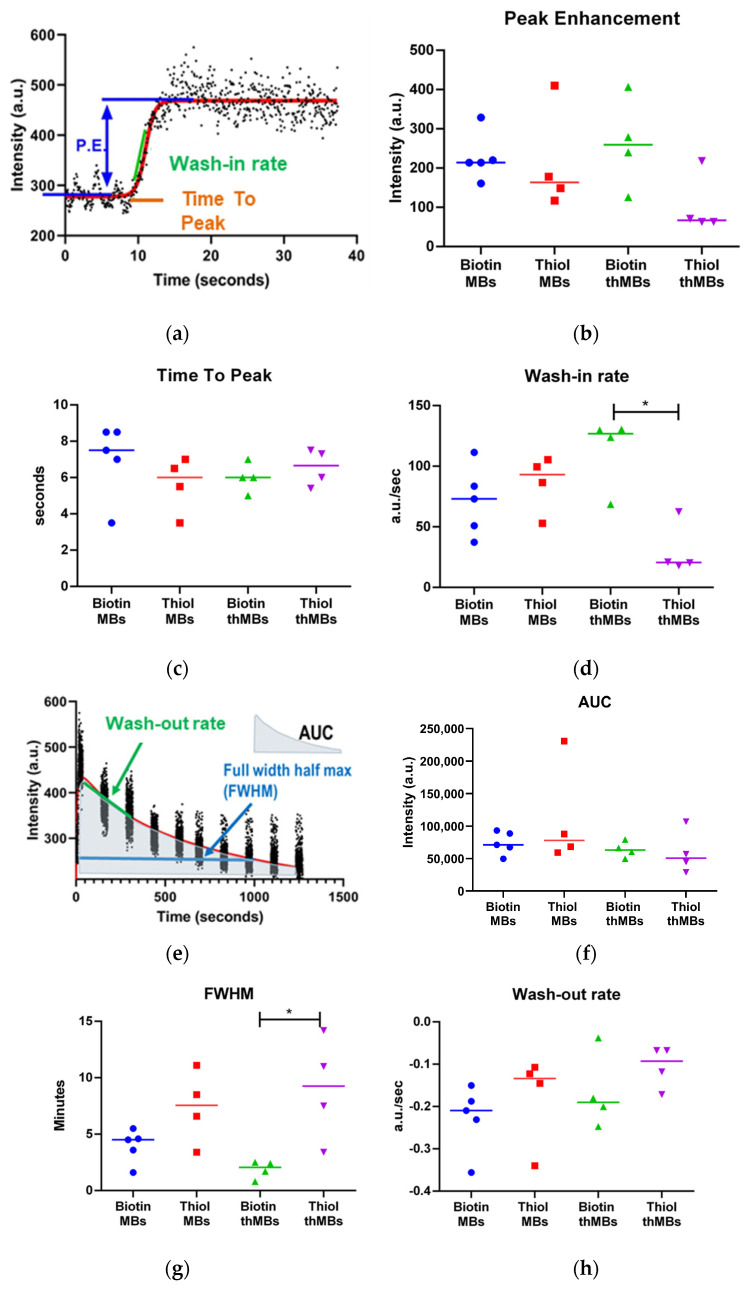
In vivo imaging showing subtle differences in imaging parameters between biotin–avidin and maleimide–thiol MBs and thMBs. (**a**) Parameters of the time intensity curve, generated by MB injection, were examined for differences in peak enhancement (P.E.) (**b**), time to peak (**c**) and the wash-in rate (**d**) between biotinylated or thiolated targeted MBs or thMBs. (**e**) The lifetime of the MB architectures was also examined in terms of the area under the curve (AUC) (**f**), the length of time that the contrast-enhanced signal intensity was detected at half the maximum signal or above (FWHM—(**g**)), and the wash-out rate (**h**). Individual data points represent a single mouse, the median is denoted, * *p* < 0.05, Mann–Whitney, two-tailed.

The different MB structures were examined for their ultrasound imaging properties. VEGFR2-targeted MBs and thMBs were then injected intravenously into the tail vein of mice whilst imaging the aorta using high-frequency ultrasound. Multiple in vivo imaging and lifetime parameters were derived from the injection loop video (Figure 7a–d) and the subsequent 20 min of imaging videos (Figure 7e–h). No statistically significant differences were seen between MBs with biotinylated antibodies or thiolated antibodies in their imaging or lifetime. For biotin or thiol-containing thMBs, peak signal enhancement (Figure 7b), time to peak enhancement (Figure 7c), the area under the curve (Figure 7f) and the decay rate/wash-out rate (Figure 7h) were all not significantly different. However, the wash-in rate of the thiol-containing thMBs was slower than that of the biotin thMBs and the FWHM, which is a measure of lifetime of the MBs was longer (Figure 7d,g). These data indicate that the thiol thMBs were slower to enter the aorta but lasted longer in vivo than biotin thMBs.

**Figure 8:**
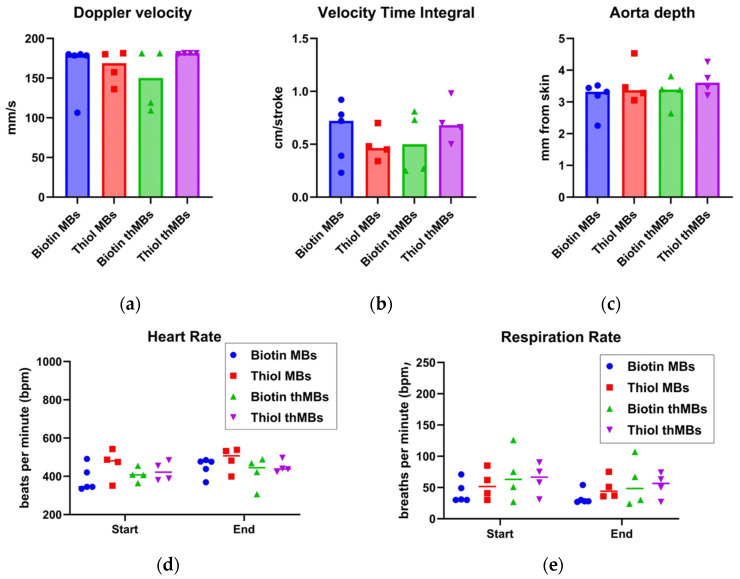
In vivo imaging differences in biotinylated and thiolated MB architectures are not due to differences in physiology between animals. (**a**) Doppler velocities (mm/s) were measured per animal using pulsed wave Doppler signal in the aorta alongside velocity time integral (cm/stroke, **b**), depth of the aorta (mm, **c**), mean heart rate at the beginning and end of the imaging process (beats per minute, bpm, **d**) and the same for the respiration rate (bpm, **e**). The results are shown for individual mice per MB type. ANOVA followed by Sidak’s multiple comparisons showed no statistically significant differences between the MB types for any of the physiological parameters measured.

To determine whether these differences in vivo imaging properties were related to physiological differences between mice, the imaging depth of the aorta was measured, the peak positive Doppler velocity and the velocity time integral (how far the blood travels within a flow period) were determined. The mean heart and respiration rates from the injection video loop and the mean heart and respiration rates from the final video loop (20 min after injection) were also examined—for any differences that may account for the changes in imaging parameters seen. No statistically significant differences were seen in any of the groups (Figure 8a–e).

**Figure 9:**
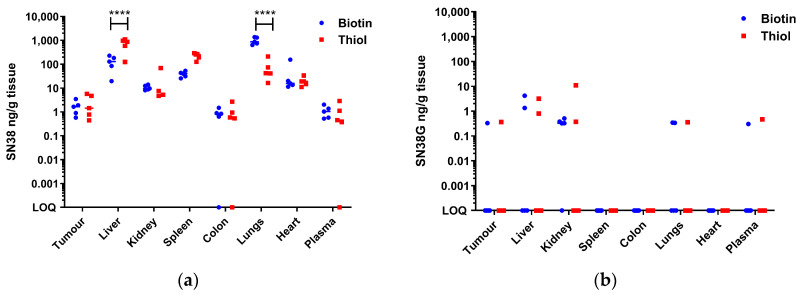
In vivo drug delivery of the two MB architectures showing differences in biodistribution. LC-MS/MS quantitation of tumour and organs from mice injected with thMBs that contained either biotin–avidin or maleimide–thiol linkages. The concentration of the encapsulated drug, SN38 (**a**), and its glucuronidated metabolite, SN38G (**b**), was determined in each tissue. *n* = 5 mice per group with the median denoted. Two-way ANOVA followed by Sidak’s multiple comparisons is shown **** *p* < 0.0001. LOQ means the sample was below the limit of quantitation (10 pg/mL for both SN38 and SN38G).

In terms of drug delivery to tumours and biodistribution, mice were injected with either biotinylated or thiolated thMBs and subjected to an ultrasound pulse at the tumour site. Tumours and organs were collected one hour after injection. The results show no difference in drug delivery to the tumour with either structure (Figure 9). However, there was a significant difference in the biodistribution where more thiolated thMBs accumulated in the liver than biotinylated thMBs but less in the lungs. SN38G, the inactive glucuronide of SN38, was quantifiable at this time point in some samples, indicating the release of the drug from the liposomes and its subsequent glucuronidation prior to elimination. No differences were seen in SN38G biodistribution, giving an indication that the release rates from the liposomes/uptake of the free drug after ultrasound-mediated cavitational release were similar for both thMB structures.

## 4. Discussion

Development of therapeutic microbubbles for drug delivery to humans is a challenging area particularly with regard to clinical manufacturing. Currently, clinical trials involving MB-assisted drug uptake use a co-delivery mechanism whereby the therapeutic agent and MB are administered sequentially. We have shown that direct bonding of the therapeutic agent to the MB and targeting this to tumours can enhance circulatory times and reduce non-specific delivery of the therapeutic agent [7]. However, the biotin–avidin linkages may not be the most advantageous for fast clinical translation. 

Here, we show how maleimide–thiol linkages show equivalent targeting of MBs in vitro to biotin–avidin linkages. In imaging terms, VEGFR2-targeted MBs showed equivalent image enhancement parameters. Targeting ratios were of a similar order of magnitude to those seen by Bam et al. using a Thy1-targeted MB with maleimide–thiol linkages [23] for imaging pancreatic ductal carcinoma. Much better targeting ratios were observed by the same group using an affibody to target cells expressing B7-H3 protein. These were linked to Horizon-generated MBs using maleimide–thiol and showed 100-fold better targeting to B7-H3-expressing cells in vitro than non-expressing cells [24]. Clearly there is much scope for improving the targeting moiety to enhance both imaging and thMB delivery over and above standard antibodies.

For thMBs, the biotin–avidin linked versions showed an enhanced wash-in rate in vivo but were not as long lasting as maleimide–thiol linked versions (although we acknowledge the spread of the data in this group). It was not apparent why these subtle differences occurred from looking at physiological parameters and studies are ongoing to look at the physical characteristics of the thMBs to determine any differences that could account for these. To our knowledge no other group has studied the exact same MB structures in parallel to compare to our data. 

Both types of linkages were safe in terms of haemolysis, so small amounts of TCEP or neutravidin are not causing any detrimental effects upon injection, and all mice were equally able to easily tolerate these MBs. Differences were observed, however, in terms of drug delivery from the thMBs in a xenograft tumour model. The delivery and metabolism of the drug was equivalent, but the maleimide–thiol linkage thMBs showed reduced uptake in the lung, concomitant with an increase in liver uptake compared to their biotin–avidin counterparts. Redirection to the liver should be advantageous as thiol groups are expected to oxidise more rapidly than biotin–avidin and are therefore excreted faster. This should minimise off-site residency times of any thMBs. Longer time scales would be interesting to study to determine whether this is the case.

Maleimide–thiol linkages have been described as unstable in vivo since the reaction is reversible, and much effort has been directed at making this linkage irreversible [25]. Shedding of payloads from thiosuccinate containing antibody drug conjugates (ADCs) in plasma are 50–75% within 7–14 days [26,27]. In this application, the linkages in a thMB would not need to be stable for longer than one hour at most since any gas-filled microbubble will have dissipated by this point in vivo. Indeed, the application of an ultrasound trigger that bursts the thMBs at the site of tumour occurs after only 4 min of circulatory time, and we have shown that this greatly improves tumour volume reduction over just thMB delivery alone [7]. In addition, the VEGFR2 target is on the tumour vasculature so much more easily accessible to vascular contrast agents compared to antibody drug conjugates that need time to extravasate from blood vessels deep into tissue. However, any free-flowing liposomes attached to VEGFR2 antibody (and lipids from a dissipated liposome) may require longer-term linkages to allow further uptake via endocytosis and other methods that would have rapid clinical translation are being investigated [28]. Although this study has shown equivalence of thMB binding in vitro, this could be further defined in vivo using flash-replenishment studies [7]. In addition, it would be interesting to elucidate why differences were observed in the thMB lifetime studies (decreased wash-in rate but increased lifetime) by examining the physical properties of the thMBs themselves potentially by using a technique such as atomic force microscopy.

## 5. Conclusions

This study compared two thMBs that differed in the linkages used to attach both drug-encapsulated liposomes and antibodies to the microbubble. Both biotin–avidin and maleimide–thiol linkages were examined for their ability to specifically bind to VEGFR2, for their ability to bind liposomes and for their ability to bind to target cells in vitro. In addition, their ultrasound imaging properties and drug delivery capability was examined in vivo. Both linkages showed equivalence in production of thMB structure, and the in vitro specificity of binding and safety profiles. In vivo imaging showed some subtle differences for thMBs, wherein biotin thMBs had a faster wash-in rate than thiol thMBs, but thiol thMBs were longer-lived. Drug delivery to tumours was also equivalent, but interestingly, thiol thMBs altered the biodistribution and/or elimination route of thMBs away from the lungs and towards the liver compared to biotin thMBs. This may have implications for developing clinical applications of thMBs using these linkages and warrants further in-depth pharmacokinetic studies to elucidate these differences further.

## Figures and Tables

**Table 1:** Characterisation of biotinylated liposomes or thiol-functionalised liposomes. Three preparations of each liposome were generated and the amount of SN38 encapsulated was quantitated by absorption spectroscopy. The mean diameter and concentration of liposomes generated was determined by nanoparticle tracking analysis. N.D., not determined.

Type	Input (μg/mL)	Encapsulated (μg/mL)	Mean Diameter (nm)	No. Liposomes (×10^12^/mL)
Biotin	400	360	264	3.02
Biotin	400	353	323	3.11
Biotin	400	240	N.D.	N.D.
Mean	400	318	294	3.1
Thiol	400	374	199	8.1
Thiol	400	382	289	4.42
Thiol	400	361	193	6.24
Mean	400	372	227	6.3

**Table 2:** Characterisation of liposome-loaded MBs generated using biotin–avidin or maleimide–thiol linkages. Five preparations of each type of liposome-loaded MB were generated and the concentration and size of these was analysed by optical imaging.

Type	Liposome-Loaded MBs (×10^8^/mL)	Mean Diameter (μm)
Biotin	4	1.6
Biotin	7.48	1.5
Biotin	4.44	1.2
Biotin	2.49	2.4
Biotin	2.3	2.3
Mean	4.1	1.8
Thiol	5.05	1.5
Thiol	7.09	1.7
Thiol	5.99	1.7
Thiol	1.66	2.4
Thiol	0.61	2.6
Mean	4.1	2.0

## Data Availability

The raw data supporting the conclusions of this article will be made available by the authors on request.
